# Feasibility study of using high-throughput drug sensitivity testing to target recurrent glioblastoma stem cells for individualized treatment

**DOI:** 10.1186/s40169-019-0253-6

**Published:** 2019-12-30

**Authors:** Erlend Skaga, Evgeny Kulesskiy, Marit Brynjulvsen, Cecilie J. Sandberg, Swapnil Potdar, Iver A. Langmoen, Aki Laakso, Emília Gaál-Paavola, Markus Perola, Krister Wennerberg, Einar O. Vik-Mo

**Affiliations:** 1 Vilhelm Magnus Laboratory for Neurosurgical Research, Institute for Surgical Research and Department of Neurosurgery, Oslo University Hospital, P.O. Box 4950, Nydalen, 0424 Oslo, Norway; 2 Institute of Clinical Medicine, Faculty of Medicine, University of Oslo, P.O. Box 1112, Blindern, 0317 Oslo, Norway; 3 Institute for Molecular Medicine Finland, FIMM, University of Helsinki, Tukholmankatu 8, 00290 Helsinki, Finland; 4 Department of Neurosurgery, Helsinki University Hospital and Clinical Neurosciences, University of Helsinki, Topeliuksenkatu 5, 00260 Helsinki, Finland

**Keywords:** Glioblastoma, Recurrent glioblastoma, Glioblastoma stem cells, High-throughput drug screening, Drug sensitivity and resistance testing, Individualized medicine, Drug sensitivity

## Abstract

**Background:**

Despite the well described heterogeneity in glioblastoma (GBM), treatment is standardized, and clinical trials investigate treatment effects at population level. Genomics-driven oncology for stratified treatments allow clinical decision making in only a small minority of screened patients. Addressing tumor heterogeneity, we aimed to establish a clinical translational protocol in recurrent GBM (recGBM) utilizing autologous glioblastoma stem cell (GSC) cultures and automated high-throughput drug sensitivity and resistance testing (DSRT) for individualized treatment within the time available for clinical application.

**Results:**

From ten patients undergoing surgery for recGBM, we established individual cell cultures and characterized the GSCs by functional assays. 7/10 GSC cultures could be serially expanded. The individual GSCs displayed intertumoral differences in their proliferative capacity, expression of stem cell markers and variation in their in vitro and in vivo morphology. We defined a time frame of 10 weeks from surgery to complete the entire pre-clinical work-up; establish individualized GSC cultures, evaluate drug sensitivity patterns of 525 anticancer drugs, and identify options for individualized treatment. Within the time frame for clinical translation 5/7 cultures reached sufficient cell yield for complete drug screening. The DSRT revealed significant intertumoral heterogeneity to anticancer drugs (p < 0.0001). Using curated reference databases of drug sensitivity in GBM and healthy bone marrow cells, we identified individualized treatment options in all patients. Individualized treatment options could be selected from FDA-approved drugs from a variety of different drug classes in all cases.

**Conclusions:**

In recGBM, GSC cultures could successfully be established in the majority of patients. The individual cultures displayed intertumoral heterogeneity in their in vitro and in vivo behavior. Within a time frame for clinical application, we could perform DSRT in 50% of recGBM patients. The DSRT revealed a remarkable intertumoral heterogeneity in sensitivity to anticancer drugs in recGBM that could allow tailored therapeutic options for functional precision medicine.

## Background

Glioblastoma (GBM) is a devastating form of cancer. Despite multidisciplinary treatment, the tumor almost invariably recurs within 9 months [1, 2]. For the recurrent disease, there are no treatment options documented to prolong survival, possibly leaving enrollment into a clinical trial the best treatment option [3].

The survival benefit of repeated tumor resection in recurrent GBM (recGBM) is limited [4, 5]. Subsequently, only a minority of patients with recGBM undergo secondary surgery [1].

In the newly diagnosed GBM, sphere-forming glioblastoma stem cells (GSCs) derived from tumor biopsies is a well-studied and relevant model system of the treatment-naïve disease [6–9]. There exists, however, limited data of the GSC population in recGBM [10]. The biology of the recurrent disease, which ultimately is the disease the patients succumb to, therefore remains inadequately described. In turn, this leads to a clinical translational gap, as early phase clinical trials in GBM mostly recruit patients with recurrent disease while the foundation of these new treatments almost exclusively are based on treatment-naïve primary GBM models [10].

The intricate tumor heterogeneity at the cellular and molecular level in the treatment-naïve GBM poses a substantial challenge for therapeutic progress on its own [11–14], but even more for treatment of the relapsed disease. Recent studies have described a longitudinal heterogeneity of tumor evolution following therapy, as the selection pressure exerted by standard treatment display tumor-to-tumor variability [15–18]. A fraction of relapsed tumors seems phylogenetically derived from dominant clones in the primary tumors, while others display a highly branched subclonal tumor evolution [15–17]. A minority of tumors develop a treatment-induced hypermutational phenotype at recurrence [19, 20]. Despite established tumoral and evolutional heterogeneity in GBM, clinical trials are still mostly designed to investigate treatment effects at the population level [21–23].

A therapeutic strategy to address tumor heterogeneity is matching the right drug to the individual patient using genomics-driven profiling. However, genomics-driven oncology for individualized treatments allows clinical decision making in only a small minority of screened patients. Even in the presence of a druggable oncogenic mutation the clinical applicability of targeted therapies has proven difficult [24–26]. Individualized treatments may also be constructed using functional approaches by automated drug screening technology for testing of hundreds of anticancer compounds directly to a patient’s cancer cells ex vivo [27].

We have previously described the intertumoral heterogeneity in patient-specific drug sensitivity patterns in the treatment-naïve GBM [13]. However, the feasibility of translating automated drug sensitivity testing of a patient`s cancer cells ex vivo to individualized treatment in solid tumors is immature [28]. This study therefore aimed to establish a bed-to-bench-to-bed clinical translational protocol for individualized treatment in recGBM utilizing patient-specific recGBM stem cell cultures and high-throughput drug sensitivity and resistance testing (DSRT). To evaluate feasibility, we investigated (i) the capability to propagate GSC cultures from recGBM ex vivo, (ii) how tumor heterogeneity in recGBM is reflected in drug sensitivity patterns to a large panel of anticancer drugs, and (iii) whether individualized treatment options can be suggested using automated drug screening and drug sensitivity scoring within a time frame suitable for clinical translation.

## Methods

### Brain tumor biopsies and cell cultures

Glioblastoma biopsies were obtained from ten informed patients with explicit written consent undergoing surgery for recGBM at Oslo University Hospital, Norway, as approved by The Norwegian Regional Committee for Medical Research Ethics (REK 07321b, 2017/167). The cell cultures were established both from several focal tumor biopsies and the ultrasonic aspirate generated during surgery. The cell cultures were established and maintained under tumorsphere forming conditions, as previously described [6]. Differentiation was induced, and the cells were fixed and stained, as previously described [6]. Images were acquired using Olympus Soft Imaging Xcellence software v.1.1. The total number of cells in serial passaging was quantified as previously described [13]. All experiments in this study have been performed within the 10th passage of the individual culture. Patient characteristics are summarized in Additional file 1.

### Definition of time frame of clinical protocol

In cohorts after resection of recGBM the median progression-free survival is reported ranging from 2.0 to 7.8 months [5, 29, 30]. To evaluate the feasibility of using autologous recurrent GSC cultures for individualized therapy, we defined a fixed time frame of 10 weeks from surgery to complete the entire pre-clinical work up; which included cell culture expansion (6 weeks), DSRT and data analysis (1 week), and treatment initiation (3 weeks).

### Flow cytometry analysis

Cells were suspended in PBS with 2% fetal bovine serum (Biochrom) and stained with directly conjugated antibodies (CD15-PerCP, R&D Systems, CD44-APC, Thermo Fisher Scientific, CD133-PE, Miltenyi Biotec, CXCR4-PE, Miltenyi Biotec) according to the manufacturer’s instructions. Cells were washed three times before analysis by flow cytometer LSRII (BD Bioscience). FlowJo software v.10.4.1 was used for data analysis. Dead cells were excluded by propidium iodine (Thermo Fisher Scientific), and doublets were excluded by gating.

### Intracranial transplantation

The National Animal Research Authority approved all animal procedures (FOTS 8318). C.B.-17 SCID female mice (7–9 weeks old, Taconic, Ejby, Denmark) were anesthetized with a subcutaneous injection of zolazepam (3.3 mg/mL), tiletamine (3.3 mg/mL), xylazine (0.45 mg/mL) and fentanyl (2.6 μg/mL). The cells were prepared and 2 μL of a single cell suspension containing 100,000 cells/μL was xenografted into the right striatum, as previously described [6]. The animals were regularly monitored for signs of distress and killed by cervical dislocation after 15 weeks or earlier if weight loss > 15% or neurological symptoms developed. The brains were harvested and further processed as previously described [6]. Images of brain sections were acquired using Axio Scan.Z1 (Carl Zeiss). Processing of images was performed using ImageJ 2.0.

### qRT-PCR

RNA was extracted using the RNeasy Micro Kit (Qiagen GmbH) and evaluated by Nanodrop spectrophotometer (Thermo Fisher) and Experion System (Bio-Rad). The high capacity cDNA Reverse Transcription Kit, TaqMan Fast Advanced Master Mix, TaqMan oligonucleotide primers and probes [Hs00157674_m1 (GFAP), Hs00801390_s1 (TUBB3)] and the ABI Prism Detection System and software (all from Applied Biosystems) were used according to the manufacturer’s instructions. Human β-Actin [Hs9999999903_m1 (ACTB)] was used as housekeeping gene. The thermal cycling conditions were 20 s at 95 °C, followed by 40 cycles of 1 s at 95 °C and 20 s at 60 °C. The relative gene expression levels were calculated using the standard curve method.

### Subclassification of GSC cultures

Subgrouping of the GSC cultures as proneural or mesenchymal was performed by analyzing RNA sequencing data. The library preparation was performed using the Truseq mRNA protocol according to the manufacturer and the samples sequenced on the Illumina HiSeq platform (paired end 2 × 75 bp). Normalized expression data was further analyzed in J-Express 2012. Unsupervised hierarchical clustering was performed as previously described [13].

### Drug sensitivity and resistance testing

The oncology drug collection consisted of 525 anticancer compounds and covered most U.S. Food and Drug Administration and European Medicines Agency (FDA/EMA)-approved anticancer drugs and investigational compounds with a broad range of molecular targets. 35% (184/525) of the drugs were of approved status (both oncological and non-oncological indications). 48% (252/525) of the drugs were in clinical investigational phases, and the rest (17%, 89/525) in preclinical investigations. Overview of the drug collection is provided in Additional file 2. The high-throughput DSRT was undertaken using an automated facility at the Institute for Molecular Medicine Finland, University of Helsinki, Finland, as previously described [13]. The cells were transported in L-15 (Lonza) as tumorspheres on ice. The complete drug screening required > 10^7^ cells. The patient-derived recurrent GSCs were plated at a density of 3000 cells/well in pre-drugged plates and after 72 h cell viability was measured using CellTiter-Glo^®^ Luminescent Cell Viability Assay, (Promega), as previously described [13]. The resulting data were normalized to positive (benzethonium chloride) and negative (DMSO) control wells. The quantification of drug sensitivity was utilized by the drug sensitivity score (DSS) [13, 31]. In brief, each drug was evaluated over a 5-point dose-escalating pattern covering the therapeutic range. The resulting dose–responses were analyzed by automated curve fitting defined by the top and bottom asymptote, the slope, and the inflection point (EC_50_). The curve fitting parameters were used to calculate the area defined as area of drug activity (between 10% threshold and 100% relative inhibition to positive and negative control) relative to the total area. To reduce the impact of toxic drug effects, the integrated response was divided by the logarithm of the top asymptote, as previously described [31]. The quantification of drug sensitivity was then calculated into a single measure as the DSS. The DSS ranges from 0 to 50, where higher number translates into increased drug efficacy. The selective drug sensitivity score (sDSS) of each compound was calculated using two independent reference databases; (i) our in-house GBM drug screening database (up to n = 18 GBMs tested for drug sensitivity to the drug collection) and (ii) the FIMM database of drug sensitivity to normal bone marrow cells from healthy donors (n = 5). Control samples of normal bone marrow cells were obtained from informed and consenting donors, and the sampling was approved by the institutional ethics committee, as previously described [27, 32]. The DSS from which the GBM reference database is based upon is available from previous reports by [13] us together with the additional files presented in this study. The sDSS was quantified as the difference between DSS in the individual sample to the average DSS of GBMs (denoted sDSS_GBM_), and as the difference between DSS in the individual sample to the average DSS of normal bone marrow cells (denoted sDSS_BM_). The selection of reference database is stated in the text when the analysis was applied.

### Temozolomide treatment

Cells were plated at 5000 cells/well in a 96-well plate (Sarstedt), cultured for 24 h before adding TMZ. The efficacy of TMZ was evaluated over a 5-point dose-escalating pattern covering the therapeutic range (therapeutic range: 5.0–50 µM, test range 0.4–250 µM) [33]. Cell viability was assessed after 10 days of incubation using Cell Proliferation Kit II XTT (Roche) solution, according to the manufacturer’s instructions. The absorbance was analyzed on a PerkinElmer EnVision. The resulting data were normalized to positive (sepantronium bromide) and negative (DMSO) control wells. The resulting dose-response was analyzed by the DSS, as stated above.

### Statistical considerations

Data analysis and graphic presentation were undertaken using GraphPad Prism 8.0, J-Express 2012 (Molmine), Keynote 9.0.2, Microsoft Excel 14.7.3 and R. Correspondence analysis of drug responses and evaluation of the tumorsphere subgrouping were performed using J-Express 2012 (Molmine). Unsupervised hierarchical clustering and heat maps were generated using J-Express 2012, GraphPad Prism 8.0, and R. Statistical analysis of the overall drug sensitivity between cultures was performed using non-parametric one-way ANOVA of ranks with Kruskal–Wallis test. Correction for multiple comparisons was done by Dunn’s test. The correlation analyses were performed using Spearman correlation (ρ). A p-value < 0.05 was considered significant.

## Results

### Recurrent GBM patient characteristics

The recurrent GSC (recGSC) cultures were established from ten patients undergoing surgery for recGBM. 9/10 patients had completed standard-of-care treatment for primary GBM consisting of surgery followed by concomitant radiotherapy (RT) and chemotherapy with TMZ and thereafter adjuvant TMZ. One primary tumor (T1532) was at the time of diagnosis classified as an anaplastic oligoastrocytoma, IDH^wt^ without 1p/19q loss, that following the recent updated classification of gliomas now would be considered an anaplastic astrocytoma with molecular features of GBM. This patient underwent surgery followed by RT before recurrence of a GBM. One patient (T1615) was included at the third surgery. The median time from primary surgery to tumor relapse was 11.9 months (range: 5.1–23.6). All relapsed tumors were unifocal and recurred within 2 cm of the resection cavity from the primary surgery. Postoperative MRI demonstrated five complete and five subtotal resections with minimal contrast enhancing residual tumor volume. The clinical characteristics are provided in Additional file 1.

### Preclinical characterization of autologous recurrent GSC cultures

**Fig. 1:**
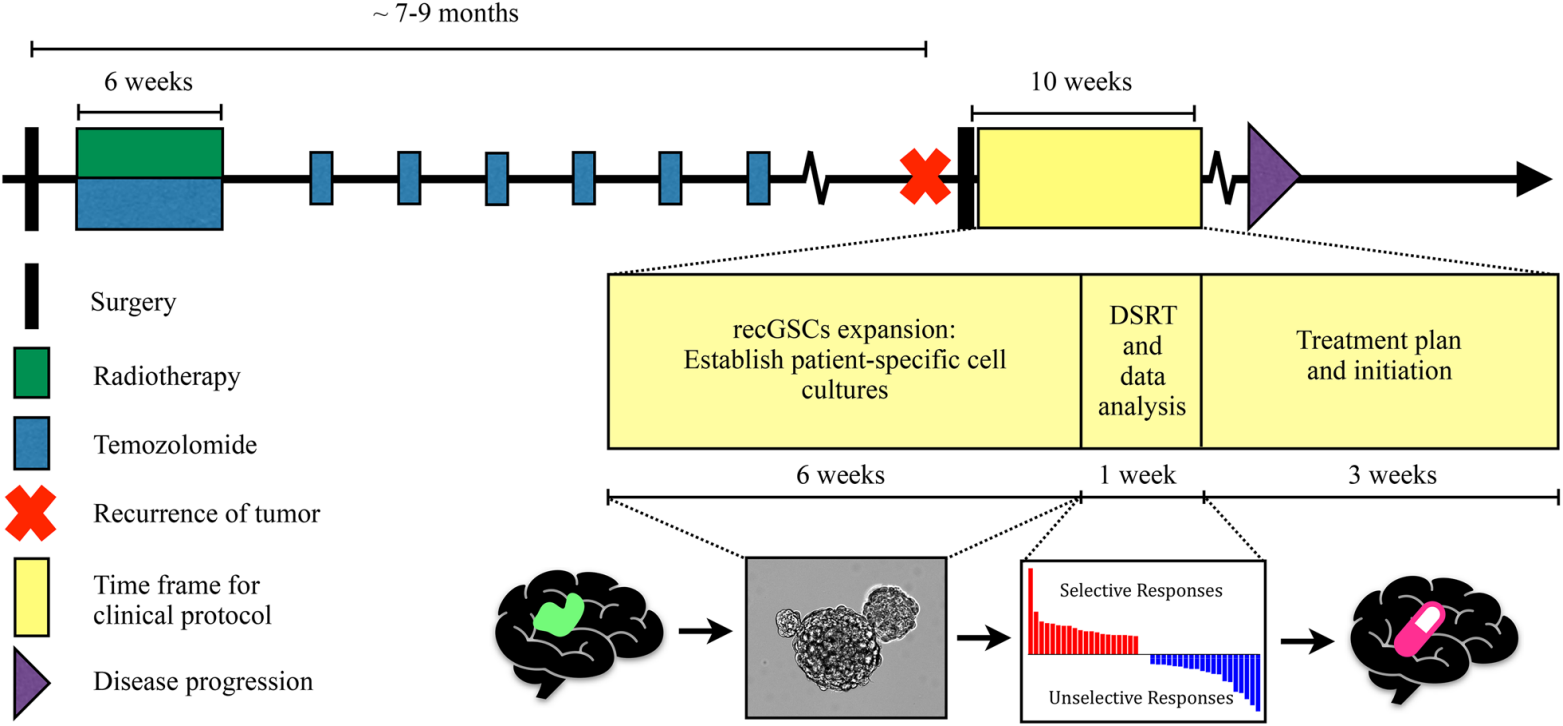
Course of the disease and time frame for clinical protocol. Glioblastoma patients typically undergo surgery followed by combined radio- and chemotherapy for 6 weeks and thereafter monthly adjuvant chemotherapy. Despite this multimodal treatment the disease almost invariably recurs within 9 months. The time frame for this clinical protocol was defined as 10 weeks following surgery for recurrent GBM, which included expansion of individualized GSC cultures for 6 weeks, automated high-throughput drug screening and data analysis for 1 weeks and scheduling a treatment plan and initiation within 3 weeks

To allow for a wide drug screen, the DSRT required 10^7^ cells. For translation to patient treatment within 10 weeks, the cell yield had to be sufficient within 6 weeks of culturing (the time frame is schematically outlined in Fig. 1). Seven of the ten cultured recGBM biopsies could be maintained for > 10 serial passages, with six forming tumorspheres and one (T1608) proliferating with adherent morphology. Three samples could not be maintained in culture. Of the seven samples capable of long-term culturing, five reached the pre-determined cell yield within 6 weeks.

**Fig. 2:**
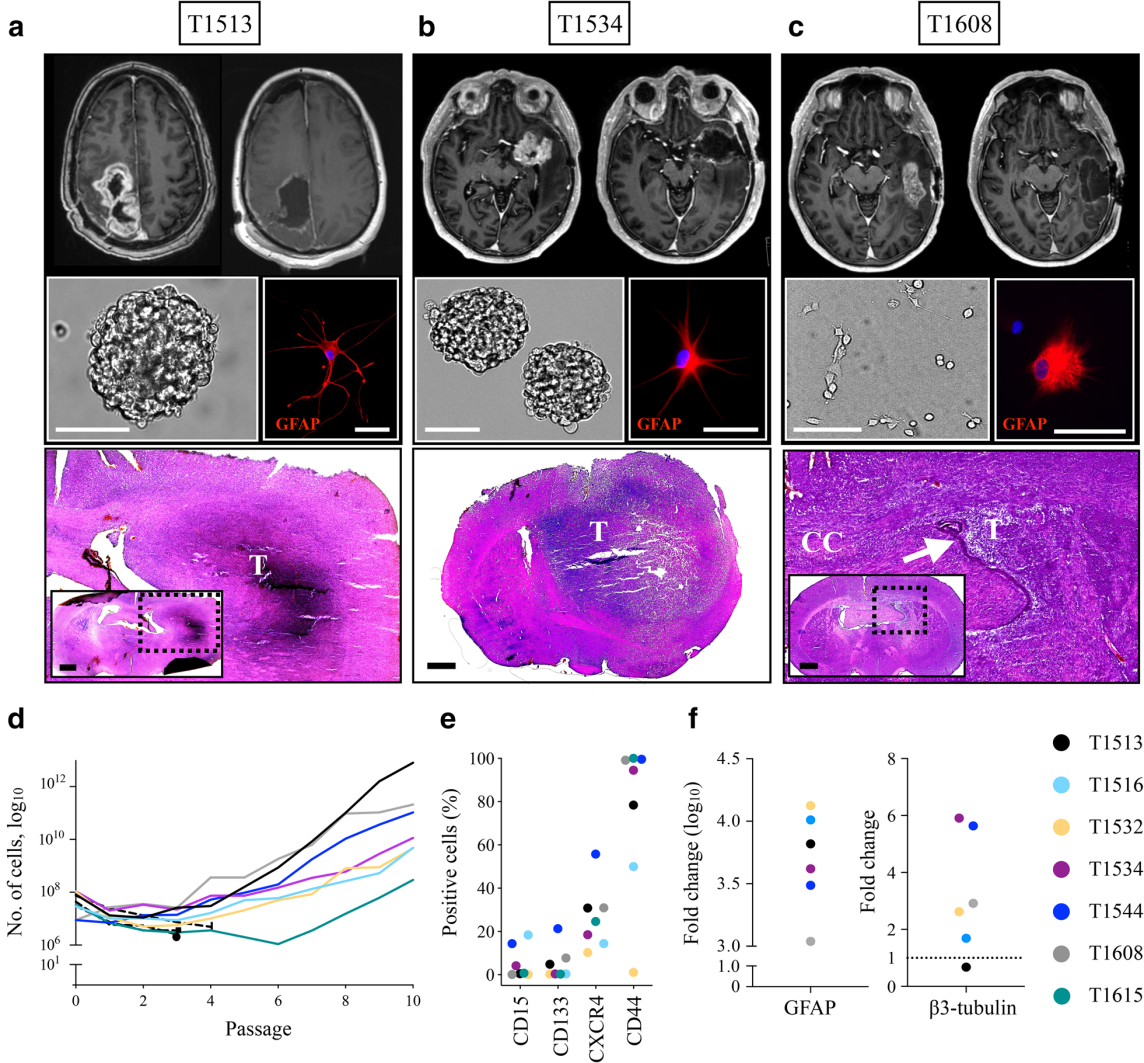
Characterization of glioblastoma stem cells from recurrent GBM. **a**–**c** Pre- and post-operative T1-weighted, contrast-enhanced MRI of three recurrent GBM with the corresponding sphere-, cellular- and xenograft morphology. The individual cultures displayed extensive tumor-to-tumor heterogeneity in their in vitro morphology (e.g. adherent growth in T1608, various differentiation morphology) and in their induced tumor phenotype (e.g. mainly bulk formation In T1534, mainly invasive in T1608). Arrow points to compressed lateral ventricle. Xenografts stained with hematoxylin & eosin. In the recGSC cultures the tumors were harvested after 15 weeks following xenografting. **d** Total cell yield following serial passages revealed intertumoral variability in their capacity for cell proliferation. Dashed lines represent tumors that could not be serially expanded. **e** Intertumoral heterogeneity in the expression of stem cell related markers evaluated by flow cytometry. **f** Upon differentiation all cultures evaluated increased their expression of glial lineage marker GFAP, and all but one (T1513) increased the expression of the neuronal lineage marker β3-tubulin. Scale bar in the light microscopy images: 100 µm. Scale bar in fluorescent images 20 µm. Scale bar in brain sections 1 mm. *T* tumor, *CC* corpus callosum

To explore stem cell properties of the individual cultures, the recGSC cultures with the capacity for long-term self-renewal (n = 7) were characterized by functional assays (Fig. 2, Additional file 3). The individual cultures maintained their unique spheroid or adherent morphology upon serial passages (Fig. 2a–c, additional file 3). Within 15 weeks of grafting to immunodeficient mice, invasive intracranial tumors were formed in 6/7 recGSC cultures. The degree of tumor bulk formation and invasive pattern, however, displayed considerable tumor-to-tumor variability (Fig. 2a–c, Additional file 3). Further intertumoral differences were observed in the expression of GSC markers, the total cell yield following serial passaging, in vitro differentiation morphology and in their ability to express glial and neuronal lineage specific markers upon differentiation (Fig. 2a–f, Additional file 3). Similar to GSC cultures from treatment-naïve GBM [13], the recGSC cultures displayed intertumoral heterogeneity in their in vitro and in vivo morphology, while preserving their individual traits, thus representing an individualized model of its parent tumor.

Within the time frame of 6 weeks for expansion, five of seven recGSC cultures had sufficient cell numbers to undergo DSRT. For broader evaluation of drug sensitivity patterns in recGBM, one additional culture (T1532) underwent further culturing to reach adequate cell numbers for DSRT. Median passage number at the time of screening was 4.5 (range: 1–6).

### Intertumoral heterogeneity in drug sensitivity in recGBM

**Fig. 3:**
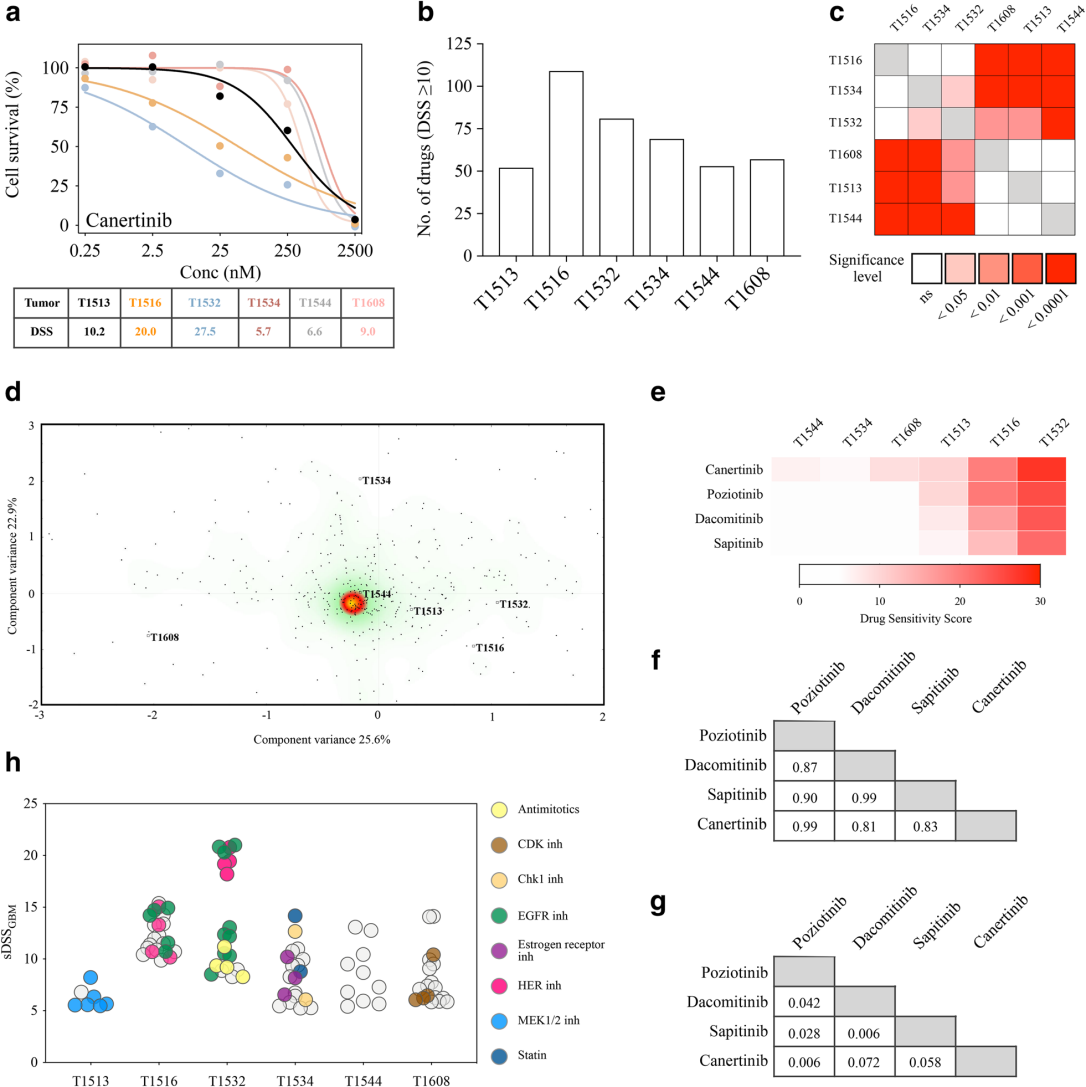
Heterogeneity in drug sensitivity in recGSCs. **a** Dose–response curves of the pan-HER inhibitor canertinib display the variation in drug efficacy in the recGSC cultures. Three responses are classified below the threshold for moderate activity (DSS ≥ 10). **b** Distribution of the number of drugs displaying a DSS ≥ 10 across the recGSC cultures. **c** Using a non-parametric one-way ANOVA of ranks, a significant difference was observed in the overall drug sensitivity across the cultures (p < 0.0001). According to the individual culture’s sensitivity to the entire drug collection (n = 525 drugs), they separated into two major clusters as the most and least sensitive. **d** Clustering of recGSC cultures by correspondence analysis based on all drug responses (n = 525) in all tumors (n = 6). The dots in the scatter plot represents the drugs in the DSRT and the color shading represent a heat map of where the average of the data is located. The scattering of tumors in the plot display both how they differ from the average and how tumors cluster together based on similarities in drug sensitivity patterns. **e** In the DSRT there were four pan-HER inhibitors that displayed a DSS ≥ 10 in recGSC cultures. **f** The consistency of T1532 being the most sensitive and T1544 the most resistant displayed an excellent correlation in correlation matrices (Spearman, ρ). **g** p-values in the correlation matrix of the pan-HER inhibitors. **h** Selecting for drugs with at least moderate efficacy (DSS ≥ 10) and increased patient-selectivity (sDSS_GBM_ ≥ 5) the distribution of individual classes of drugs with selective efficacy revealed a considerable tumor heterogeneity in drug sensitivity in recGSC cultures

We next explored whether tumor heterogeneity in recGSC cultures is reflected in the drug sensitivity to anticancer drugs using automated high-throughput technology. We have previously defined a DSS ≥ 10 as the threshold to classify a drug response as moderate to strong [13]. In total 148 drugs (28% of the drug collection) displayed this response in the recGSC culture cohort. The median was 63 drugs (range: 52–109) (Fig. 3a, b). Similar to the treatment-naïve disease [13], the sensitivity to any given drug was heterogeneous as 55% (82/148) of drugs with a DSS ≥ 10 displayed intertumoral differences equal to a moderate to strong drug sensitivity (∆DSS ≥ 10, DSS_max_ − DSS_min_). The overall sensitivity to the entire drug collection (n = 525) significantly differed between the cultures (p < 0.0001), and according to overall drug sensitivity the cultures were separated into two major clusters as the most (T1516, T1532, T1534) and the least (T1513, T1544, T1608) sensitive cultures (Fig. 3c). Correspondence analysis of the drug responses to the entire drug collection (n = 525 drugs) spread the cultures (n = 6) along the first component variance (25.6%) according to similar patterns in drug sensitivity. The clustering clearly separated T1516 and T1532 away from T1608. The second component variance (22.9%) clearly separated one culture (T1534) away from the others, however, we could not identify a shared pattern of the clustering.

We have previously reported on the biological consistency of the GSC model system in preserving individual drug sensitivity and resistance patterns [13]. The same consistency was found in cultures derived from the recurrent disease (Fig. 3e–g, Additional files 4, 5). For instance, among pan-HER inhibitors (n = 4), T1532 was the most sensitive culture to all inhibitors within that class (Fig. 3e). Correlation matrices displayed an excellent correlation (Spearman, ρ) with an average ranked correlation (± standard deviation) of pan-HER inhibitors of 0.90 (± 0.08, Fig. 3f, g). The similar pattern was found for drug resistance. While being the most sensitive to pan-HER inhibitors, T1532 was found to be the most resistant culture to CDK inhibitors. The correlation matrices displayed an average correlation (± standard deviation) of 0.82 (± 0.15) (Additional file 4). These patterns confirmed the findings from the treatment-naïve disease that individual drug sensitivity and resistance patterns are consistent in the patient-derived GSC culture model.

We next evaluated the overall heterogeneity in patient-specific drug sensitivity according to classes of drugs. Selecting drugs based on DSS ≥ 10 (above moderate efficacy) and sDSS_GBM_ ≥ 5 (high patient-specificity), we found a remarkable heterogeneity in the individual recGSC cultures sensitivity to different classes of anticancer drugs. The drugs comprised a variety of classes and mechanistic targets including conventional chemotherapies (antimitotics), hormone therapies (estrogen receptor inhibitors), metabolic modifiers (statins) and several different kinase inhibitors (CDK-, Chk1-, EGFR-, HER-, and MEK1/2 inhibitors) (Fig. 3h).

**Fig. 4:**
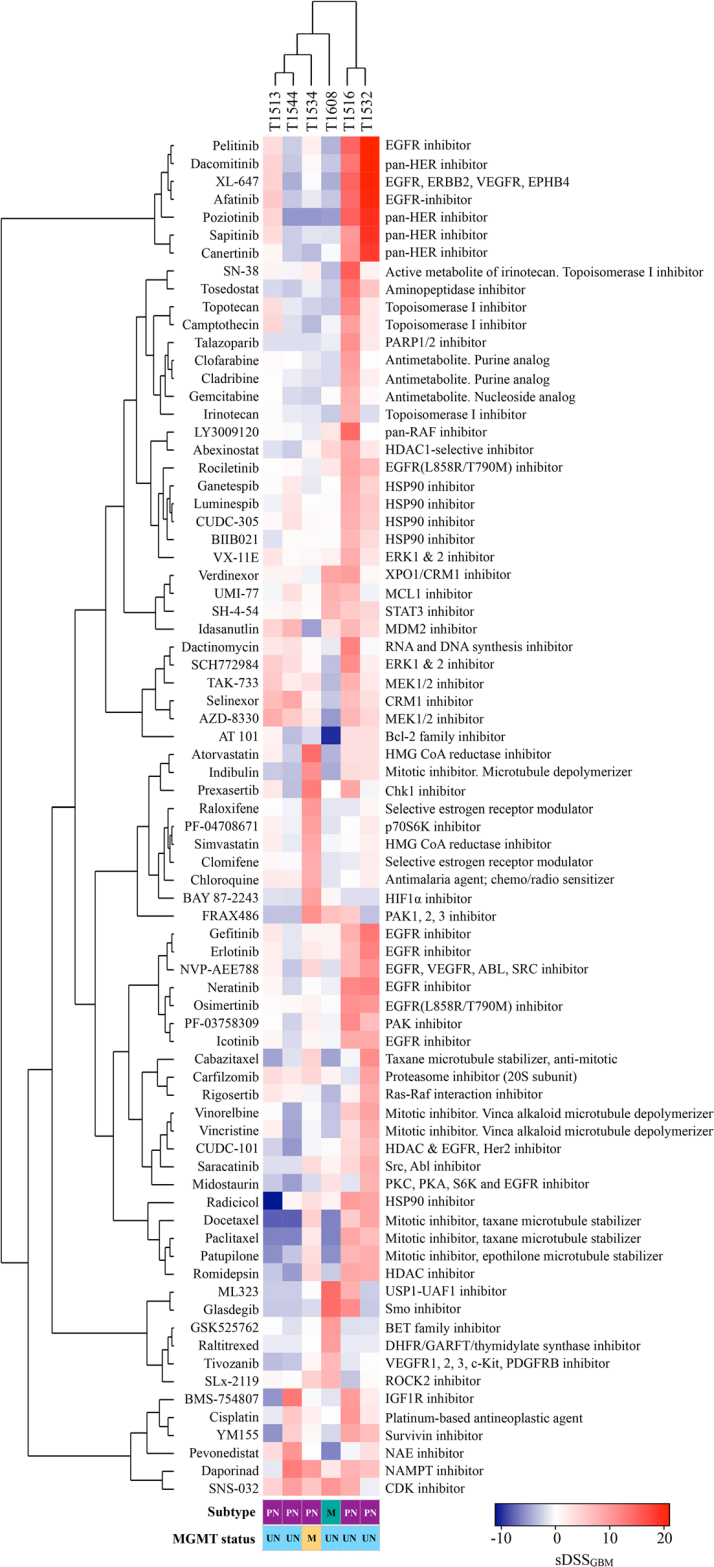
Unsupervised hierarchical clustering of drug sensitivity patterns in recGBM. Heat map and unsupervised hierarchical clustering of patient-specific drug responses (sDSS_GBM_) with Euclidian distance (cultures and drugs). The heat map is filtered by DSS ≥ 10 and sDSS ≥ or ≤ 7 (n = 76 drugs). *PN* proneural, *M* mesenchymal, *UN* unmethylated MGMT promoter, *ME* methylated MGMT promoter

After establishing differences in overall drug sensitivity and in individual drug sensitivity patterns in the recGSC culture cohort, unsupervised hierarchical clustering of selective drug responses (by sDSS_GBM_) revealed that the relationship of cultures with similar drug sensitivity patterns was linked to the mechanistic target of the drugs (Fig. 4, Additional files 6, 7).

#### Sensitivity to temozolomide in recGSCs

As 6/7 of the recurrent GBM patients had been treated with TMZ, the recGSCs should in principle be TMZ-resistant. This could be used to evaluate the validity of the drug screening. In the DSRT none of the recGSC cultures displayed any sensitivity to TMZ (DSS = 0 in all cultures, Additional file 5). To evaluate sensitivity to alkylating agents in clinically relevant concentrations, we and others have previously reported that cell viability assays require longer incubation for adequate evaluation [34, 35]. We therefore performed additional cell viability assays of 10 days incubation using clinically relevant concentrations in the seven recGSC cultures. None of the cultures displayed any sensitivity to TMZ corresponding to at least moderate efficacy. The median DSS was 1.2 (range: 0.0–6.1). The resistance to TMZ was linked to MGMT gene promoter status. 6/7 cultures were classified as MGMT promoter unmethylated (cut off < 10%), while one culture (T1534) was 79.5% MGMT promotor methylated but still TMZ resistant at recurrence (Additional file 8).

#### Functional precision medicine for individualized therapy in recurrent GBM

**Fig. 5:**
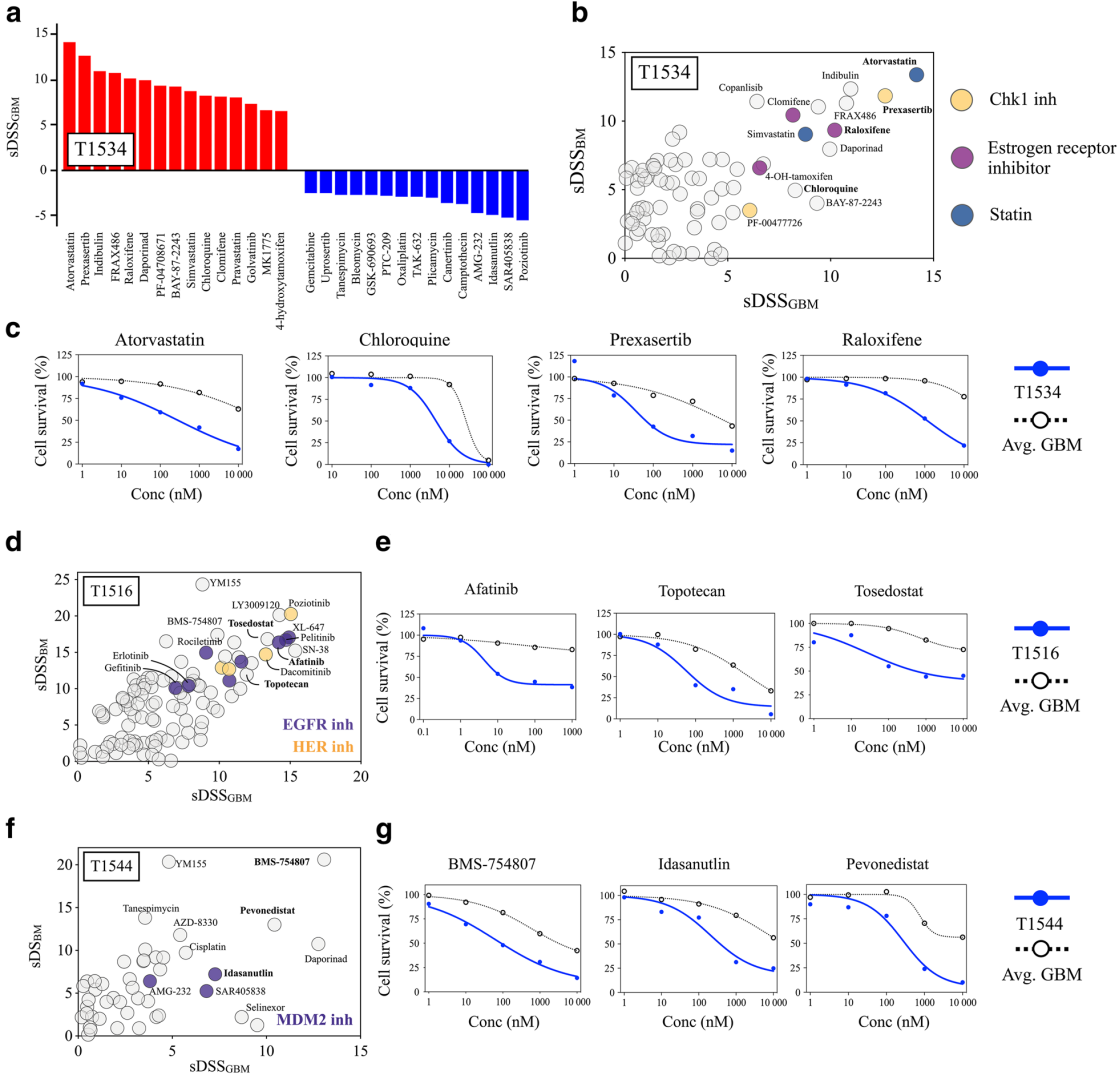
Individual therapeutic options in recGSC cultures. **a** Waterfall plot of the 15 most (red) and 15 least selective (blue) drug responses in T1534 by sDSS_GBM_. The plot displays the sensitivity to e.g. statins and estrogen receptor inhibitors, and the resistance to MDM2 inhibitors (SAR405838, AMG-232, Idasanutlin). **b** Dot plot of sDSS in T1534 using both the GBM (x-axis) and healthy bone marrow (y-axis) reference databases. Classes (color coded) and single drugs with patient-specific activity in T1534 are highlighted. **c** The corresponding dose–response curves of selected drug responses in T1534. **d**, **e** Similar dot plot and selected dose–response curves in T1516. T1516 displayed a remarkable sensitivity to EGFR- and HER-inhibitors, of which several with approval status available for fast translation. **f**, **g** Dot plot and selected dose-response curves in T1544. T1544 was among the least sensitive tumors, and displayed an increased sensitivity to MDM2-inhibitors, that currently are evaluated in clinical trials of GBM (NCT03158389)

The heterogeneity in drug sensitivity in recGBM was found in the individual cultures at the level of overall sensitivity, in sensitivity to specific drug classes, and in sensitivity to individual drugs with specific mechanistic targets. The DSRT could therefore identify individualized treatment options in each patient-derived recGSC culture. For individualized therapy selection, we focused on compounds that exhibited at least a moderate drug response (DSS ≥ 10) combined with a selective drug response (sDSS) to the individual patient (Fig. 5a). For patient-specific drug response evaluation, we utilized our in-house reference databases of drug sensitivity (database of drug sensitivity in (i) GBM and (ii) bone marrow cells from healthy donors) to quantify the differential responses (Fig. 5b).

The DSRT revealed remarkable intertumoral heterogeneity in drug sensitivities for individualized therapy (Fig. 5, Additional file 9). For instance, T1534, which was found as one of the most sensitive cultures to the entire drug collection, and clearly separating along the second component variance in correspondence analysis, had highly patient-specific responses to a diverse range of drug classes including checkpoint kinase 1 inhibitors, estrogen receptor inhibitors and statins (Fig. 5b, c). Additionally, single drug responses from a range of classes and mechanistic targets were identified, e.g. indibulin (conventional chemotherapy), daporinad (metabolic modifier), chloroquine (antimalarial drug/autophagy inhibitor), BAY 87-2243 (HIF1 inhibitor) and several different kinase inhibitors (the PI3K-inhibitor copanlisib and the PAK-inhibitor FRAX486). Several of the patient-specific drug responses were from drugs of approved status (e.g. atorvastatin, chloroquine, clomifene, copanlisib, raloxifene, simvastatin) with potential for fast clinical translation.

We identified effective (DSS ≥ 10) and selective (with both sDSS_GBM_ and sDSS_BM_ ≥ 5) anticancer drugs with approved status in all recGSC cultures (Fig. 5b–g, Additional files 10, 11). The number of drugs of approved status differed among the cultures, demonstrating that selection of drugs for patient treatment is more complex in certain tumors. Further highly selective drug responses (DSS ≥ 10 and both sDSS_GBM_ and sDSS_BM_ ≥ 10) of either approved drugs or drugs in clinical developmental phases were found in 5/6 recGSC cultures that underwent DSRT, suggesting potential for patient-specific therapy options with highly selective drugs for functional precision medicine (Fig. 5, Additional file 9).

## Discussion

This study demonstrates the feasibility of a bed-to-bench-to-bed clinical translational protocol in recGBM utilizing automated drug screening and autologous recGSC cultures for individualized therapy selection. From surgical biopsies in recGBM, we were able to expand GSCs form 70% of the patients and generate adequate cell numbers from 50% within a clinically acceptable time period. The DSRT revealed patient-specific drug vulnerabilities to single drugs, as well as classes of drugs, with the potential for fast clinical translation. The sensitivity profiles covered a range of drug classes and molecular targets that demonstrated a remarkable intertumoral heterogeneity in drug sensitivity patterns in recGSCs.

The complex heterogeneity in GBM represents a substantial challenge for therapeutic progress. The heterogeneity in primary GBMs have widely been described at the cellular [9], and molecular [11, 12, 14] level, resulting in a heterogeneous clinical picture evident by interpatient differences in tumor growth kinetics [36], response to current therapies and survival [37]. Recent studies have looked into the biology of the relapsed disease and added new dimensions of heterogeneity by describing tumor-to-tumor variations in evolutionary dynamics of treatment resistant tumor cell populations [15–17]. This translates into a complex intertumoral heterogeneity at the time of recurrence of GBM. Consequently, targets identified based on analyses of the of the untreated disease may not be informative in treating the heavy-pretreated and relapsed GBM. As expected, we found intertumoral heterogeneity in patient-derived recGSC cultures in their proliferative capacity, phenotype of xenograft (tumor bulk and invasion patterns), and patient-specific sensitivities to anticancer drugs. Together, this supports the notions that treatment strategies in recGBM should be individualized and must be translated from preclinical studies using material and models from recGBM.

The natural course of recGBM leads to rapid disease progression and clinical deterioration. This makes it challenging to establish a direct clinical translational protocol utilizing time-consuming generation of individualized cell cultures. Inclusion following this protocol is inherently limited to patients eligible for secondary surgery. It has been estimated that up to 30% of recGBM are accessible to undergo secondary surgery [3, 4]. Such estimates, however, are primarily based on studies with a retrospective design. In cohorts of recGBM patients enrolled in clinical surgical trials the fraction of patients undergoing secondary surgery are reported to be considerably higher [2, 5]. This protocol further relies on the ability to successfully establish cell cultures. The success rate has been reported to be as low as 30% and 25% in primGBM and recGBM, respectively [38]. In an operating series, we have previously reported a success rate of establishing cell cultures in > 70% of first surgery GBM biopsies [7], which compares similarly to the success rate in establishing individual cell cultures from recGBM in this study. However, as we defined a time frame of 6 weeks for cell culture establishment and proliferation this further selected 50% of the total patient cohort eligible for treatment. There are, however, some adjustable variables in this protocol. The extent of DSRT can be adjusted by reducing the number of drugs (e.g. 17% of the drugs are in preclinical development not available for fast clinical translation). The number of drugs can also be customized to the individual cell culture according to the total cell yield for faster DSRT to allow for a higher fraction of patients to be screened at a lower complexity.

The fixed time frame of 10 weeks for this protocol may seem rigid. After performing 6 weeks of cell culturing and 1 week for DSRT and analysis, this leaves 3 weeks for treatment planning and implementation. The time period was chosen to evaluate the feasibility of finalizing the preclinical work-up for clinical translation in a heterogeneous recGBM population. We acknowledge that some patients experience disease progress within 10 weeks [39]. We may, however, reach enough cells before 6 weeks leading to faster DSRT. Similarly, in patients where the recGSC expansion is slower, the time frame for cell culturing can be expanded if the patient does not experience detrimental disease progression. The preclinical work-up can thus be adjusted for each patient for optimal arrangement.

In our DSRT we found patient-specific sensitivity to a wide range of FDA-approved drugs across different mechanistical classes, such as topoisomerase I inhibitors (e.g. irinotecan, topotecan), EGFR-inhibitors (e.g. afatinib, erlotinib), and estrogen-receptor inhibitors (e.g. clomiphene, tamoxifen). Drugs from these classes have previously been investigated in clinical trials in GBM [40–46]. Overall, the effectiveness of these drugs appears very limited, but usually cases of partial and complete responses are reported. Such responses suggest a heterogeneous pattern in drug sensitivity among patients in clinical trials. In support of clinical variation in drug sensitivities in GBM, the standard-of-care with TMZ display various effectiveness in patients. Sensitivity to TMZ can be predicted by methylation status of the MGMT-promoter [37], and tumors that are IDH-mutated have better survival prospects following standard-of-care treatment [47]. Importantly, however, even in IDH wild-type tumors that have an unfavorable methylation profile (unmethylated MGMT promoter), some patients respond to TMZ treatment as demonstrated by the increased fraction of patients surviving over 2 years after the introduction of TMZ [48]. We have previously reported a heterogeneity of individual drug sensitivity in the treatment-naïve disease that mirrors clinical response patterns [13]. We hypothesize that the ex vivo DSRT model system may identify patients with tumor cells with the highest susceptibility to a drug or class of drugs. However, it is important to consider the limitations ex vivo DSRT presents for drug discovery, as a very simplified model compared to the complex biological system in a patient.

A major challenge in the treatment of tumors of the central nervous system (CNS) is the ability to reach adequate concentrations of the drug within both the tumor and brain parenchyma across the blood–brain barrier (BBB). Penetrability and brain tissue concentrations of anticancer agents are for the vast majority of drugs, including the drugs in this study, unfortunately unknown [49, 50]. Selected compounds, such as alkylating agents (e.g. TMZ), nucleoside analogs (e.g. gemcitabine), topoisomerase inhibitors (e.g. topotecan), and a few kinase inhibitors (e.g. gefitinib, erlotinib), have been evaluated for brain penetrability with varying results [50]. A major limitation in most human studies addressing BBB penetrability is the indirect measurement of brain concentrations levels using cerebrospinal fluid (CSF) concentrations as a surrogate evaluation [50]. Thus, the true concentrations of brain parenchyma levels for the vast majority of anticancer drugs, including TMZ, are inadequately described. Evidence of brain penetrability of anticancer drugs can, however, also be inferred from clinical trials reporting tumor responses in neuro oncological disorders, such as in primary brain tumors or CNS metastases [43, 51, 52]. Examples include osimertinib in CNS metastases from non-small cell lung cancer [52] and trametinib in metastases from malignant melanoma [53]. The DSRT revealed selective drug responses of compounds with evidence of brain penetrability (for instance afatinib [43], chloroquine [51], osimertinib [52], selinexor [54], trametinib [53], topotecan [41]) in all but one recGSC culture. Importantly, there are further emerging technologies to disrupt the BBB to enhance tissue concentrations of anticancer compounds for drugs with poor penetrability [55].

Intratumoral heterogeneity is a major challenge when applying targeted treatment strategies [56]. A single tumor biopsy involves a fraction of the total tumor volume, potentially not capturing more peripheral subclones. To maximize clonal diversity, we utilized both tumor core sampling from several focal biopsies along with the ultrasonic aspirate generated during surgery. We utilized low passage cell cultures for DSRT and confirmed the tumorigenicity of tumor cells by orthotopically xenografting to immunodeficient mice. However, the study relies on in vitro evaluation of drug sensitivity, as the time frame for clinical translation is not feasible for in vivo studies. Furthermore, the DSRT investigates only sensitivities to single compounds. We acknowledge that single compound treatment seems of limited value in recGBM patients [56]. For investigation of drug sensitivities to several hundreds of anticancer drugs the requirement of total number of cells is extensive. Additional investigation of combination therapies would vastly increase requirements of cells along with the complexity of the system, and the major limiting factor in this translational protocol is the number of cells generated in cell culture. It is, however, possible to introduce combination treatments informed by DSRT although their combined effect not have been evaluated ex vivo. For successful clinical translation of combinational therapies, the choice of therapy must be carefully undertaken, considering efficacy, biodistribution and interactions.

Although the DSRT identified effective drugs available for clinical translation in all patients, the drug response patterns were remarkably heterogeneous across the recGSC culture cohort. For instance, T1516 and T1532 displayed a high sensitivity to EGFR-inhibitors, whereas EGFR-inhibitors demonstrated very limited efficacy in the remaining tumors. EGFR is a commonly altered gene in GBM that has made it an attractive target for GBM therapy. Results in clinical trials targeting EGFR alterations, even in highly selected patients, have unfortunately presented disappointing results [22]. Translating a genomic alteration into a relevant functional inhibition in a GBM cells [57] or glioma patients [24] is, however, complex. Results from the DSRT of both the untreated disease [13] and the recurrent tumors in this study, have shown consistency in the drug sensitivity patterns across a class of drugs in the individual tumor. Thus, the understanding of biological traits involved in drug sensitivity, such as sensitivity to EGFR-inhibitors, could be further elucidated by combining DSRT with molecular profiling of the individual tumor [28]. Such integration of genomic and functional data has stratified patients with acute myeloid leukemia into a functional taxonomy [27]. A similar approach of correlating genomic profiling to drug responses could strengthen the data presented in this study to more in-depth elucidate the biology underlying drug sensitivity. The aim of the current study was, however, to explore the feasibility of using functional profiling to develop a translational clinical protocol for individualized treatment decisions in recGBM. To create functional taxonomies in GBM would require larger culture cohorts for more robust linkages. An important implication of such analyses would be the ability to create a database that relates drug sensitivity patterns to tumor genetics to identify potential therapies even when only genomic data are available. In turn, that could benefit the fraction of patients were the derived tumor biopsies not adequately proliferate to perform DSRT. It may also be of benefit to inform therapeutic strategies within a shorter time frame, as the current clinical protocol relies in the time-consuming generation of individualized cell cultures.

## Conclusions

In summary, we have established the pipeline for a translational clinical protocol targeting glioblastoma stem cells in recGBM utilizing automated drug sensitivity testing. We found an extensive intertumoral heterogeneity in sensitivity to anticancer drugs in recGBM that mirrors the clinical heterogeneity in drug sensitivity in GBM. This adds experimental evidence to why population-based treatments of targeted therapies seem of limited value in a heterogeneous GBM population. In support for fast clinical translation, we found FDA-approved drugs displaying patient-specific activity in all recGBM cultures to guide individualized treatment decisions. We will further utilize the protocol to translate ex vivo DSRT to the patient bedside for functional precision medicine, however, the protocol presented here is readily translatable to other cancers grown as tumorspheres.

## Supplementary information

**Additional file 1.** Patient characteristics. Patient characteristics of which all patient-derived recGSC cultures were obtained.

**Additional file 2.** Drug collection. The drug collection used in this study.

**Additional file 3.** Preclinical characterization of recGSC cultures. MRI, in vitro spheroid and differentiation morphology and the subsequent xenograft upon transplantation of immunodeficient mice. T = Tumor.

**Additional file 4.** Drug sensitivity in recurrent GSCs across different drug classes and molecular targets. The figure displays drug class, the drug sensitivity in recGSC cultures, and average (± SD) Spearman’s coefficient (ρ) from correlation matrices. The figure displays selected drug categories from different classes to highlight the consistency in similar drug sensitivity patterns in the individual culture to a specific class of drugs.

**Additional file 5.** Drug sensitivity scores. All drug sensitivity scores for recGSCs generated in the study.

**Additional file 6.** Heat map of DSS in all drugs and cultures. Heat map and unsupervised hierarchical clustering of absolute effects (DSS) of the entire drug collection.

**Additional file 7.** Heat map of sDSS in all drugs and cultures. Heat map and unsupervised hierarchical clustering of relative effects (sDSS_GBM_) of the entire drug collection.

**Additional file 8.** Sensitivity to TMZ and MGMT promoter methylation status.

**Additional file 9.** Individualized therapeutic options in recGSCs. Dot plot of sDSS relative to both reference libraries (GBM: x-axis, BM: y-axis) in T1513, T1532 and T1608.

**Additional file 10.** Dot plot of FDA-approved drugs with patient-specific activity in all recGSC cultures. Drugs are filtered by at least moderate efficacy DSS ≥ 10 and sDSS_GBM_ ≥ 3.

**Additional file 11.** Heat map of FDA-approved drugs. Heat map and unsupervised hierarchical clustering of relative effects (sDSS_GBM_) of FDA-approved drugs filtered by DSS ≥ 10 and sDSS_GBM_ ≥ or ≤ 3.

## Data Availability

Data from the drug screening of all recurrent GBMs are included in this published article and its additional files. All other data used in the current study are available from the corresponding author on reasonable request.

## Abbreviations

BBB: blood brain barrier
CDK: cyclin-dependent kinase
Chk1: checkpoint kinase 1
CNS: central nervous system
DSRT: drug sensitivity and resistance testing
DSS: drug sensitivity score
EGFR: epidermal growth factor receptor
FDA: U.S. Food and drug administration
GBM: glioblastoma
GSC: glioblastoma stem cell
HER: human epidermal growth factor receptor
HTS: high-throughput screening
IDH: isocitrate dehydrogenase
MEK: mitogen activated protein kinase
MGMT: *O*^6^-methylguanine-DNA methyltransferase
recGBM: recurrent glioblastoma
recGSC: recurrent glioblastoma stem cell
sDSS: selective drug sensitivity score
TMZ: temozolomide
