# Supplementary figures and images for: Feasibility study of using high-throughput drug sensitivity testing to target recurrent glioblastoma stem cells for individualized treatment
Source: Clin Transl Med. 2019 Dec 30;8:33. doi: 10.1186/s40169-019-0253-6 (PMC6937360; doi:10.1186/s40169-019-0253-6)

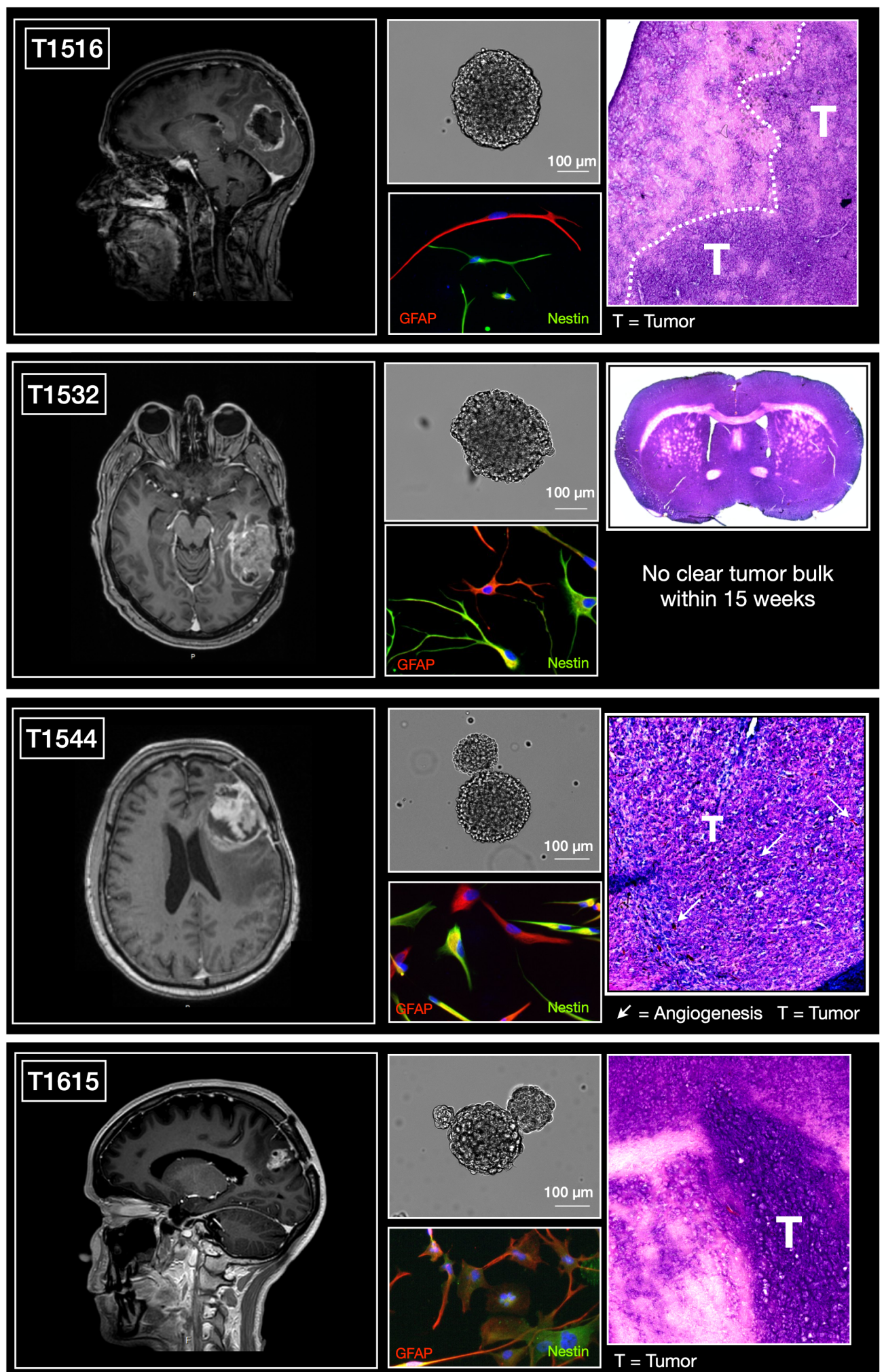

Supplement: Supplementary file 3 — Additional file 3. Preclinical characterization of recGSC cultures. MRI, in vitro spheroid and differentiation morphology and the subsequent xenograft upon transplantation of immunodeficient mice. T = Tumor. [file 40169_2019_253_MOESM3_ESM.pdf]

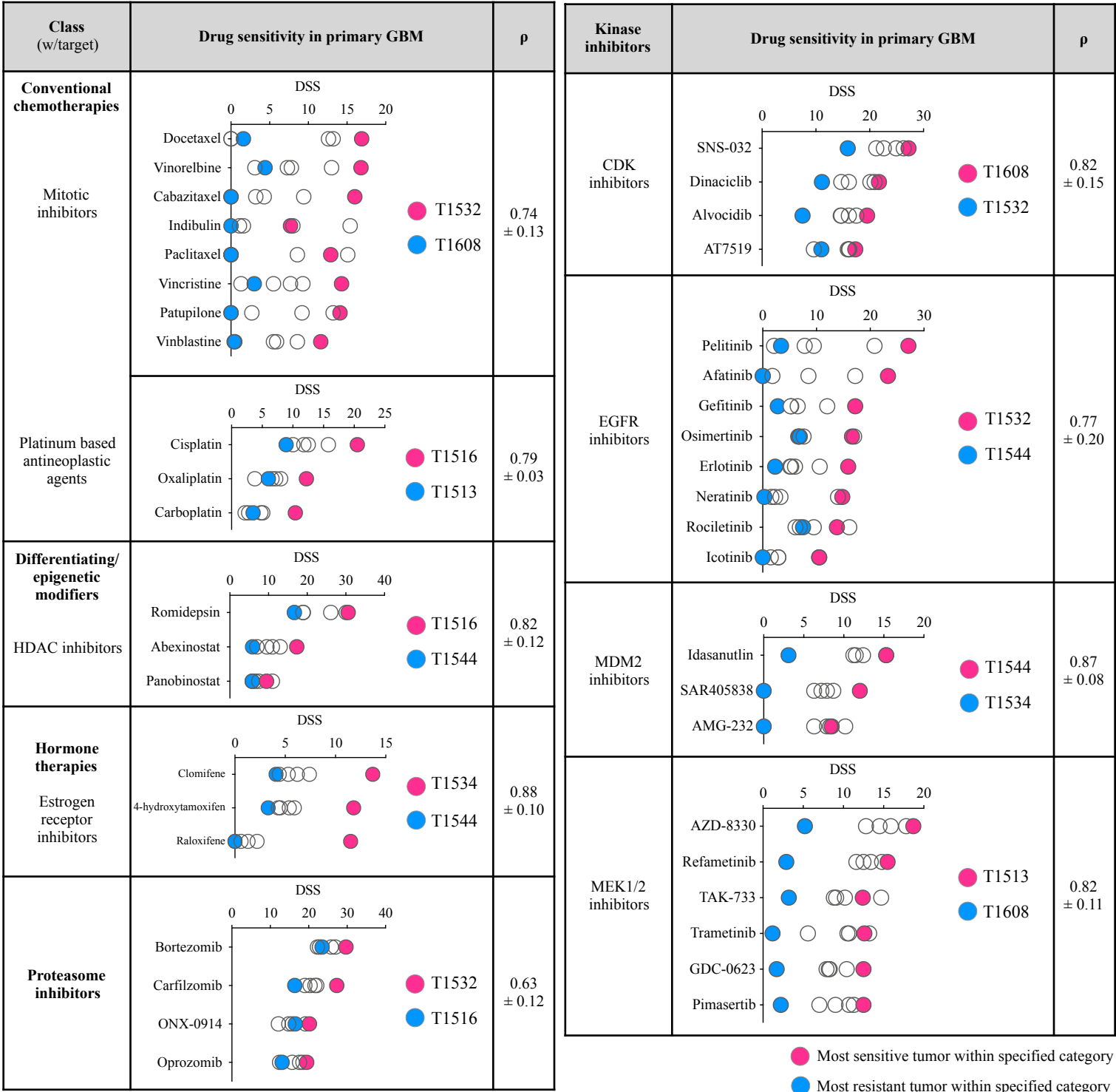

Supplement: Supplementary file 4 — Additional file 4. Drug sensitivity in recurrent GSCs across different drug classes and molecular targets. The figure displays drug class, the drug sensitivity in recGSC cultures, and average (± SD) Spearman’s coefficient (ρ) from correlation matrices. The figure displays selected drug categories from different classes to highlight the consistency in similar drug sensitivity patterns in the individual culture to a specific class of drugs. [file 40169_2019_253_MOESM4_ESM.pdf]

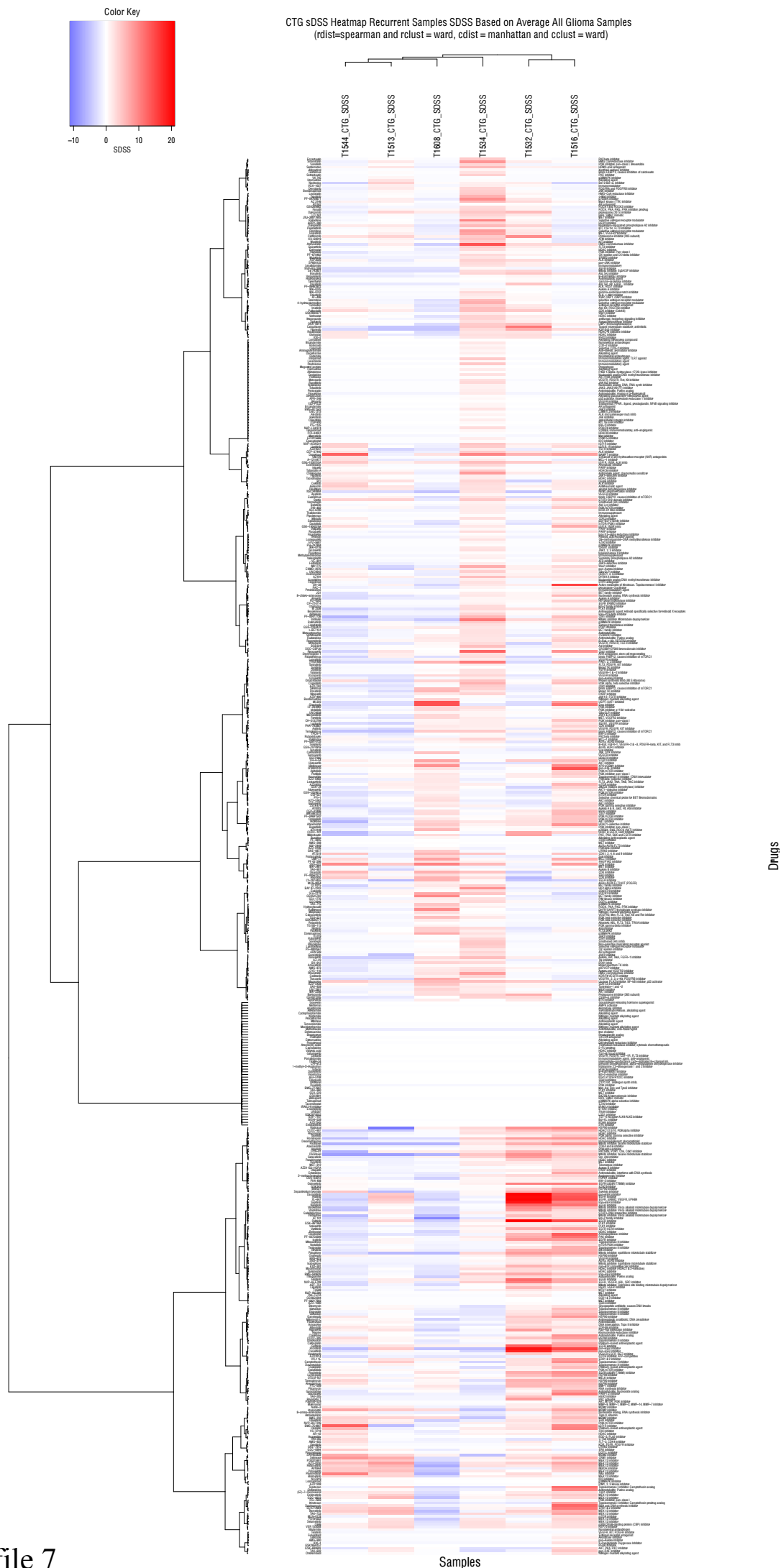

Supplement: Supplementary file 7 — Additional file 7. Heat map of sDSS in all drugs and cultures. Heat map and unsupervised hierarchical clustering of relative effects (sDSSGBM) of the entire drug collection. [file 40169_2019_253_MOESM7_ESM.pdf]

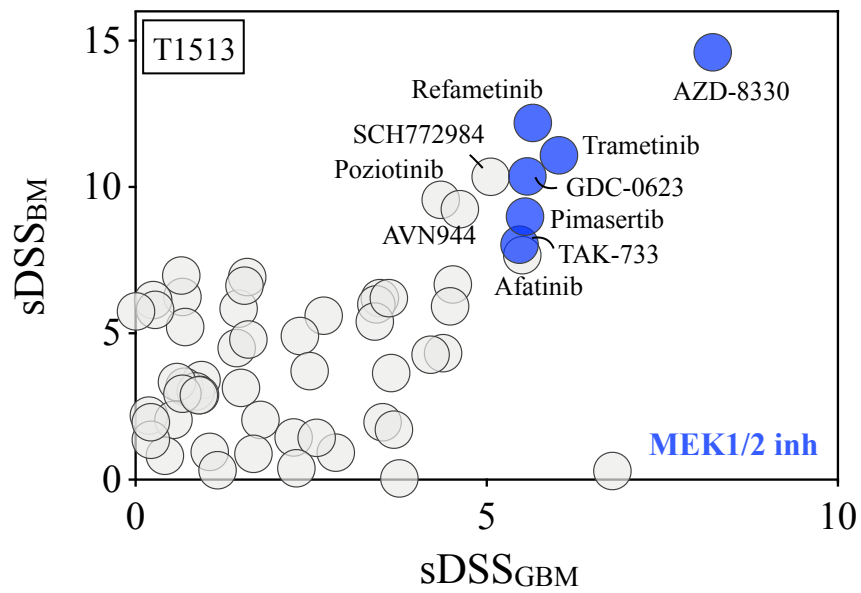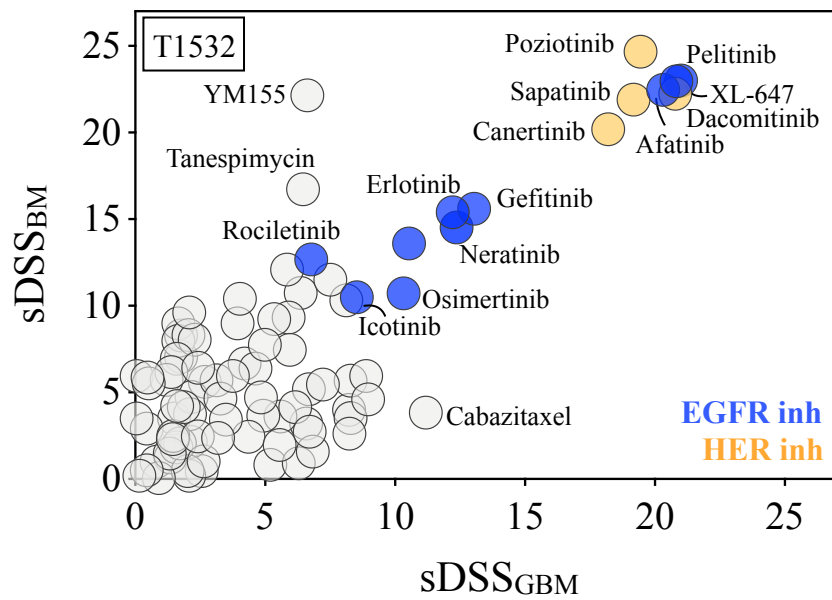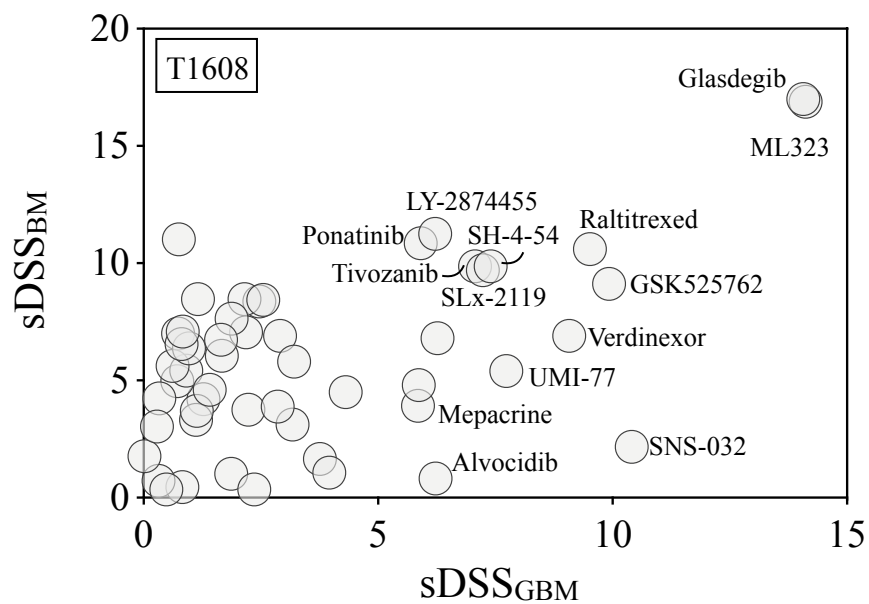

Supplement: Supplementary file 9 — Additional file 9. Individualized therapeutic options in recGSCs. Dot plot of sDSS relative to both reference libraries (GBM: x-axis, BM: y-axis) in T1513, T1532 and T1608. [file 40169_2019_253_MOESM9_ESM.pdf]

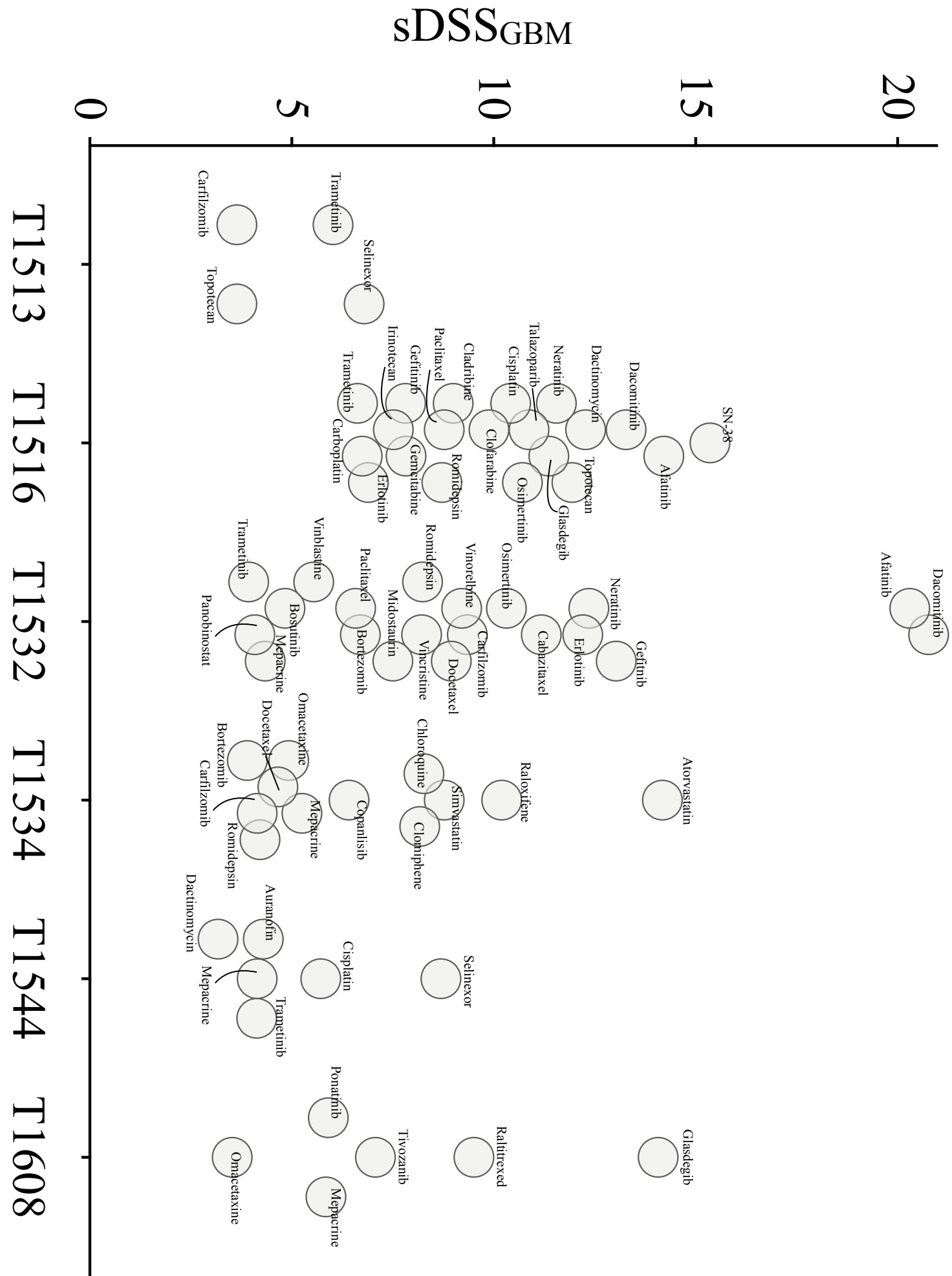

Supplement: Supplementary file 10 — Additional file 10. Dot plot of FDA-approved drugs with patient-specific activity in all recGSC cultures. Drugs are filtered by at least moderate efficacy DSS ≥ 10 and sDSSGBM ≥ 3. [file 40169_2019_253_MOESM10_ESM.pdf]

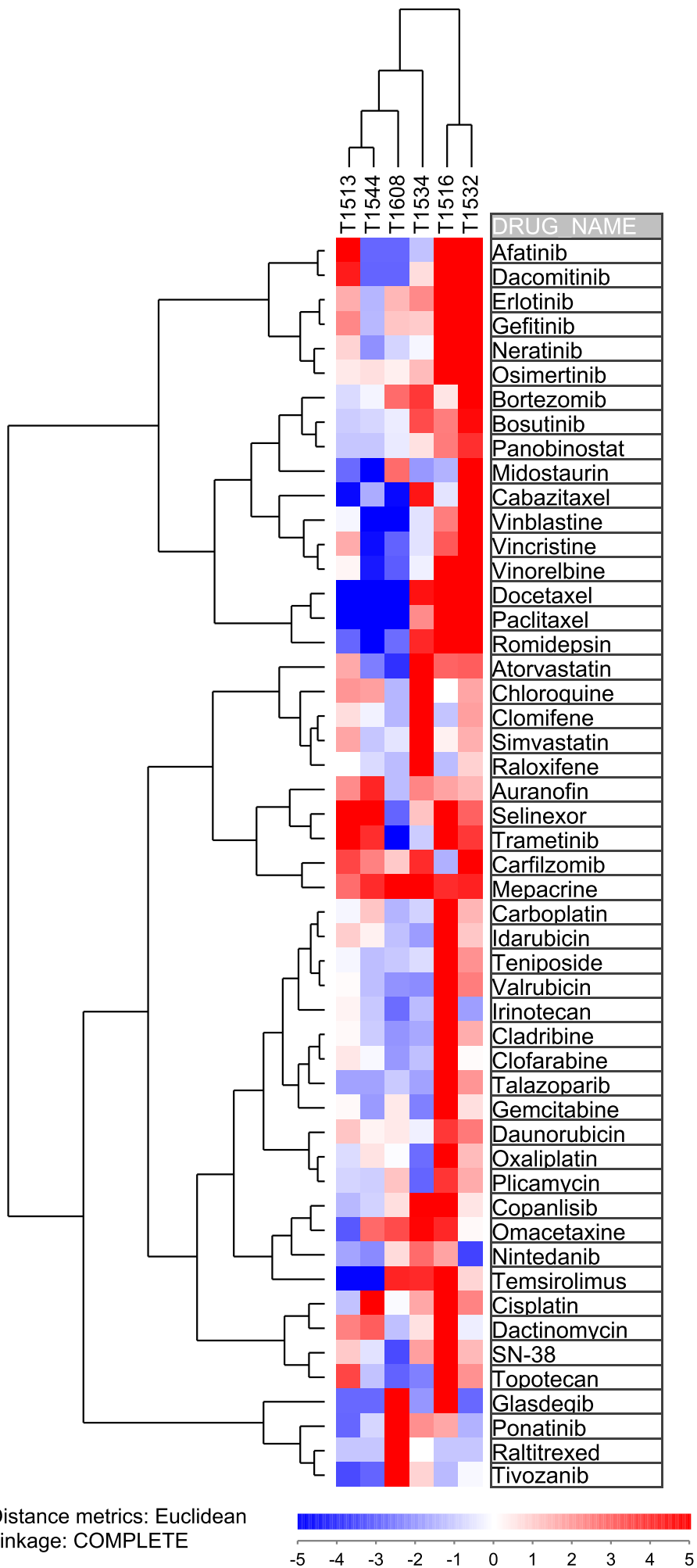

Supplement: Supplementary file 11 — Additional file 11. Heat map of FDA-approved drugs. Heat map and unsupervised hierarchical clustering of relative effects (sDSSGBM) of FDA-approved drugs filtered by DSS ≥ 10 and sDSSGBM ≥ or ≤ 3. [file 40169_2019_253_MOESM11_ESM.pdf]
