# Supplementary material for: Feasibility study of using high-throughput drug sensitivity testing to target recurrent glioblastoma stem cells for individualized treatment
Source: Clin Transl Med. 2019 Dec 30;8:33. doi: 10.1186/s40169-019-0253-6 (PMC6937360; doi:10.1186/s40169-019-0253-6)
Supplement: Supplementary file 6 — Additional file 6. Heat map of DSS in all drugs and cultures. Heat map and unsupervised hierarchical clustering of absolute effects (DSS) of the entire drug collection. [file 40169_2019_253_MOESM6_ESM.pdf]

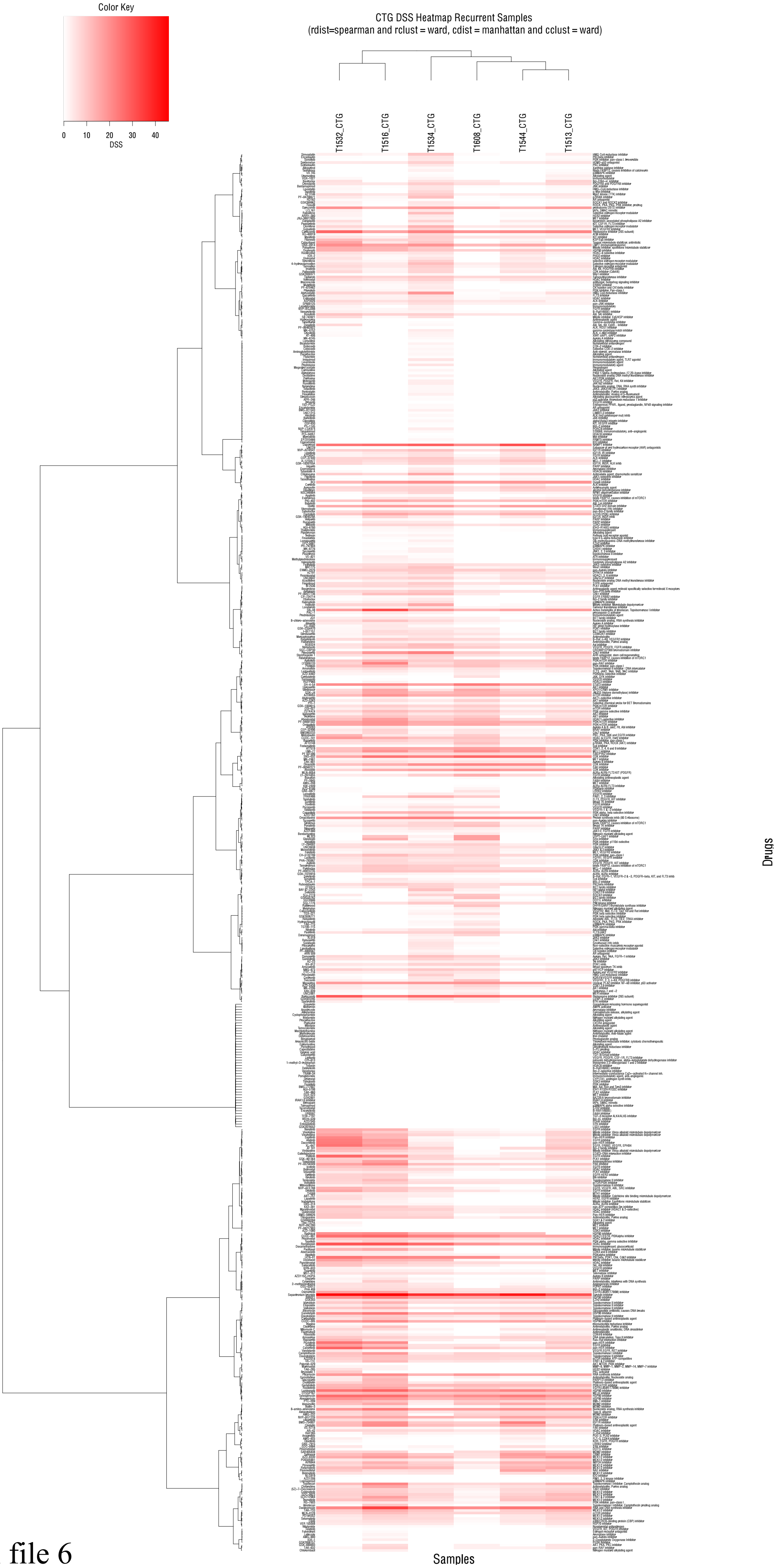

Additional file 6
